# Development of cecal volvulus after redo pancreas transplantation

**DOI:** 10.1093/jscr/rjag703

**Published:** 2026-08-14

**Authors:** Georgios Voidonikolas, Nicolas Melo, Steven A Wisel, Justin A Steggerda, Manaf Alsudaney, Tsuyoshi Todo, Irene K Kim, Todd V Brennan

**Affiliations:** Comprehensive Transplant Center, Department of Surgery, Cedars-Sinai Medical Center, Los Angeles, CA, United States; Department of Surgery, Division of Trauma and Critical Care, Cedars-Sinai Medical Center, Los Angeles, CA, United States; Comprehensive Transplant Center, Department of Surgery, Cedars-Sinai Medical Center, Los Angeles, CA, United States; Comprehensive Transplant Center, Department of Surgery, Cedars-Sinai Medical Center, Los Angeles, CA, United States; Department of Medicine, Karsh Division of Gastroenterology and Hepatology, Cedars-Sinai Medical Center, Los Angeles, CA, United States; Comprehensive Transplant Center, Department of Surgery, Cedars-Sinai Medical Center, Los Angeles, CA, United States; Comprehensive Transplant Center, Department of Surgery, Cedars-Sinai Medical Center, Los Angeles, CA, United States; Comprehensive Transplant Center, Department of Surgery, Cedars-Sinai Medical Center, Los Angeles, CA, United States

**Keywords:** cecal volvulus, large bowel obstruction, pancreas transplantation, immunosuppression

## Abstract

Cecal volvulus in recipients of abdominal organ transplants is a rare but potentially life-threatening condition that demands prompt diagnosis and intervention. Given this population's high baseline morbidity, surgical extent and complication-minimization strategies must be carefully evaluated. A 52-year-old woman with a history of brittle type 1 diabetes presented after redo pancreas transplant with abdominal pain and obstipation. She underwent a computed tomography scan, which showed mesenteric torsion of the ileocolic vessels and an air-distended cecal loop. Emergency exploratory laparotomy confirmed the diagnosis of cecal volvulus. An ileocecectomy with ileocolic anastomosis was performed. The patient recovered uneventfully and was discharged on postoperative day 5. This is the first reported case of cecal volvulus following redo pancreas transplantation. The surgical strategy must be carefully tailored to minimize risks and optimize outcomes in transplant patients.

## Introduction

Cecal volvulus is a rare and complex condition characterized by the axial twisting of the cecum, ascending colon, and terminal ileum, causing a closed-loop obstruction. It represents ~4% of all large-bowel obstructions in the United States but has a mortality rate of up to 40% [1]. The higher mortality rate is often attributed to delays in diagnosis and treatment, which may occur more commonly in transplant recipients because immunosuppression can weaken or mask symptoms.

**Figure 1:**
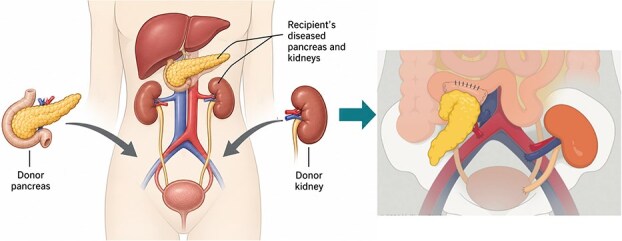
Simultaneous pancreas and kidney transplantation.

Pancreas transplantation is an established treatment for selected patients with complicated diabetes and is often performed as simultaneous pancreas–kidney transplantation, as shown in Fig. 1 [2]. Surgical exposure typically requires a midline incision and right-sided medial visceral rotation, such as a Cattell–Braasch maneuver, to access the vascular sites for anastomosis.

Pancreas retransplantation is uncommon but can have graft survival comparable to primary transplantation [3], although it is surgically challenging because the second graft is often placed ipsilaterally in the right abdomen [4].

This article presents the first reported case of cecal volvulus following redo pancreas transplantation.

## Case report

**Figure 2:**
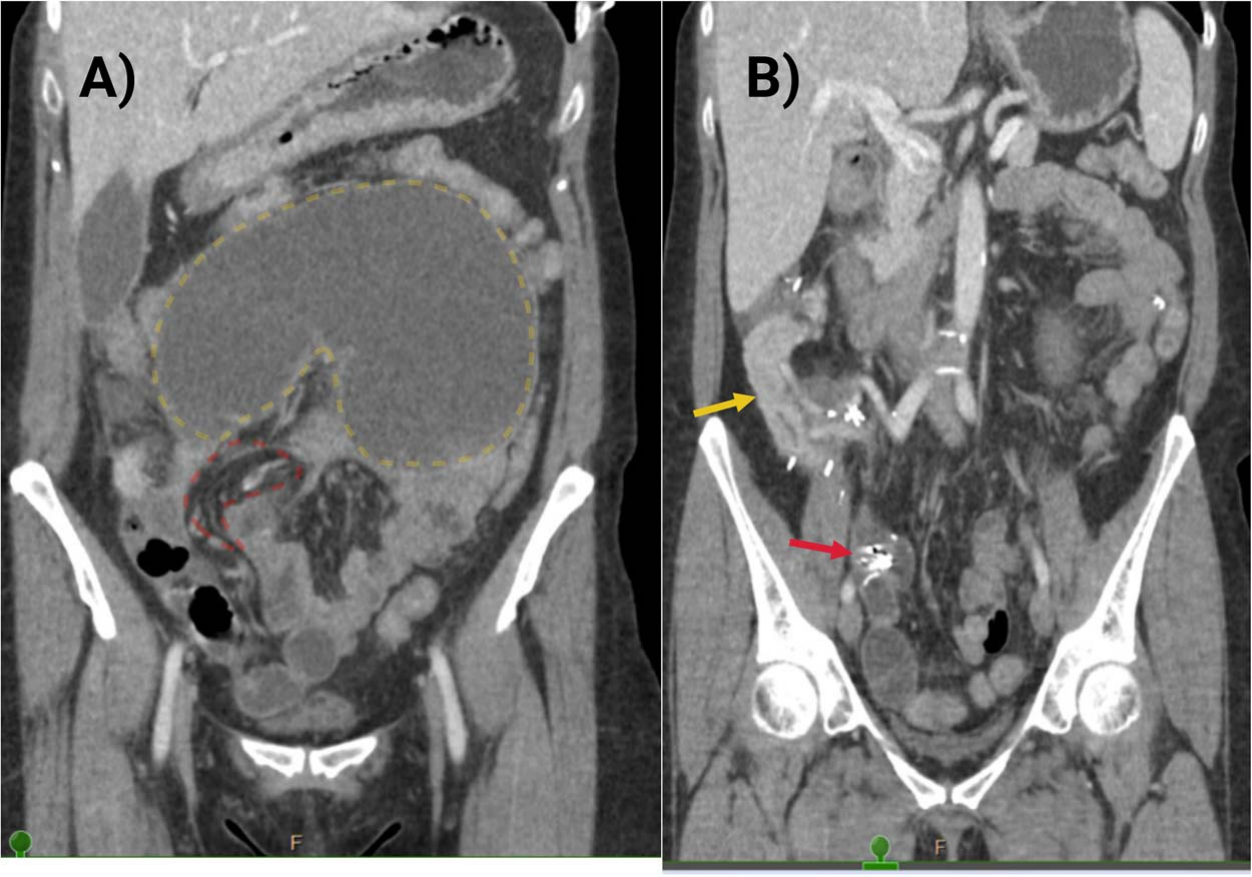
Coronal sections of intravenous contrast-enhanced CT showing (A) air-distended cecal loop/coffee bean sign, outlined by the large dashed contour, and mesenteric torsion/whirlpool sign, outlined by the smaller dashed contour, characteristic of cecal volvulus; and (B) new pancreas transplant (upper arrow) and previous pancreas transplant (lower arrow) in the right abdomen.

A 52-year-old woman with a history of brittle type 1 diabetes underwent a pancreas transplant in 2012, with the graft placed ‘tail-down’. The graft vessels were anastomosed to the right external iliac vessels, and the enteric drainage was done by a side-to-side duodenojejunostomy at 40 cm from the ileocecal valve. After 6 years, the graft failed due to rejection. Because she was HLA sensitized, she received pre-transplant rituximab, intravenous immunoglobulin, and plasmapheresis. In 2021, she underwent a second pancreas transplant above the prior graft. The second graft vessels were anastomosed to the common iliac artery and IVC, with the graft ‘tail up’. We opted to bring the new enteric anastomosis ~20 cm proximal to the previous one without taking the latter down nor creating a Roux-en-Y jejunal limb. She received anti-thymocyte globulin induction and maintenance tacrolimus, mycophenolate mofetil (MMF), and prednisone. In the following years, she reported intermittent bloating and was prescribed hyoscyamine, simethicone, and short courses of loperamide. Four years after her second transplant, she presented with 3 days of right lower quadrant pain and obstipation. She was hemodynamically stable with focal tenderness but no guarding or rebound. Laboratory evaluation showed normal white blood cell count, creatinine, lipase, and lactate, with mild metabolic acidosis. Computed tomography (CT) demonstrated mesenteric torsion of the ileocolic vessels and an air-distended cecal loop, consistent with cecal volvulus as shown in Fig. 2. A nasogastric tube was placed, and she was taken emergently for exploration.

Exploration revealed a viable but markedly dilated cecum with a torsion point near the ileocecal mesentery. Ileocecectomy with hand-sewn ileocolic anastomosis was performed because the patient was hemodynamically stable. Postoperatively, tacrolimus and prednisone were continued, MMF was discontinued, and she was discharged on postoperative day 5. Pathology showed cecal dilation with wall thinning and mucosal ischemic changes involving the ileum and cecum.

## Discussion

Cecal volvulus is an uncommon but serious cause of large-bowel obstruction, with reported incidence of 2.8–7.1 cases per million annually and female predominance [5]. It results from torsion or folding of a mobile cecum and may present with acute abdominal pain, distention, nausea, vomiting, and bowel obstruction; compromised mesenteric blood flow can lead to ischemia or perforation [1]. Symptoms typically include an acute onset of abdominal pain and signs of bowel obstruction, such as abdominal distention, nausea, and vomiting. Disruption in mesenteric blood flow can cause ischemia and potentially lead to intestinal perforation, requiring urgent surgery. Predisposition is often related to incomplete fixation of the right colon, reported in >10% of adults, with volvulus developing after additional triggers such as prior abdominal surgery, constipation, ileus, or distal obstruction [6, 7]. Postoperative cecal volvulus has been reported after extensive right-colon mobilization during laparoscopic appendectomy and after kidney transplantation or donor nephrectomy [8, 9].

It is possible our patient developed a mobile cecum following the Cattell-Braasch maneuver performed to create space for the second pancreas transplant. The patient’s periodic use of loperamide and hyoscyamine may have also caused colonic dysmotility that contributed to the volvulus. These prior and new enteric anastomoses may have created additional tether points that contributed to volvulus. Although the prior and new enteric anastomoses could theoretically create tether points for obstruction or volvulus, they were not involved in our case.

Definitive management generally requires ileocolic resection or right hemicolectomy rather than cecopexy or cecostomy [10]. Following resection of the affected the decision for primary anastomosis, diversion, or end ileostomy must balance anastomotic risk against ostomy-related morbidity, including dehydration, electrolyte abnormalities, and kidney injury [11]. In transplant recipients, immunosuppression may further influence healing; tacrolimus has been associated with fewer wound-healing complications than mTOR inhibitors, whereas chronic corticosteroids and immunosuppression may increase postoperative risk [12, 13]. Other studies of colorectal resection in the transplant population showed higher rate of mortality, and wound complications, although there was no significant difference in anastomotic leak rates when compared with immunocompetent patients [14, 15]. Given this patient’s stable physiology and healthy-appearing bowel after resection, we performed primary anastomosis and adjusted immunosuppression postoperatively.

## Conclusion

Cecal volvulus after redo pancreas transplantation is rare and requires prompt diagnosis and operative management. Prior right-sided mobilization and immunosuppression should be considered when tailoring surgical strategy and postoperative care.

## Data Availability

Deidentified data supporting the findings of this case report are available from the corresponding author on reasonable request, subject to institutional review and patient privacy restrictions.
